# Clonal Dissemination of Extended-Spectrum Cephalosporin-Resistant *Enterobacterales* between Dogs and Humans in Households and Animal Shelters of Romania

**DOI:** 10.3390/antibiotics11091242

**Published:** 2022-09-13

**Authors:** Andreea Paula Cozma, Cristina Mihaela Rimbu, Flavia Zendri, Iuliana Elena Maciuca, Dorina Timofte

**Affiliations:** 1 Department of Exact Sciences, Faculty of Horticulture, University of Life Sciences, 700490 Iasi, Romania; 2 Department of Public Health, Faculty of Veterinary Medicine, University of Life Sciences, 700490 Iasi, Romania; 3 Institute of Infection, Veterinary and Ecological Sciences, Department of Veterinary Anatomy, Physiology and Pathology, Leahurst Campus, University of Liverpool, Neston CH64 7TE, UK

**Keywords:** cephalosporin-resistance, faecal-carriage, transmission, companion animals

## Abstract

Faecal carriage of extended-spectrum cephalosporin-resistant (ESC-R) *Enterobacterales* in healthy pets is a concerning issue. This study aimed to determine the prevalence, genetic background, and potential for interspecies transmission of these bacteria between dogs and humans within the same household (HH) or shelter environment in Romania. Faecal samples (*n* = 263) collected from healthy dogs (*n* = 102), their owners (*n* = 32), as well as dogs (*n* = 110) and staff (*n* = 19) from dog shelters, were screened for ESC-R carriage. Clonal relatedness of canine and human *Escherichia coli* isolates was established using Fourier Transform Infrared Spectroscopy (FTIR), followed by Illumina WGS of selected isolates. The highest prevalence of ESC-R *Enterobacterales* faecal carriage was identified in staff working at dog shelters (78.9%), followed by dogs from households (44.11%), dog owners (43.7%), and dogs from shelters (27%). FTIR identified 15 clusters of closely related *E. coli* isolates, including dog and human isolates from the same environment. Co-carriage of ESC-R isolates in both the dog and owner was identified in 12 HHs (37.5%), with two HHs (6%) having both the owner and dog carrying isolates with identical FTIR spectra, phylogroup, resistance genes, and Inc plasmids. Major ExPEC lineages such as ST127, ST10, ST155, and ST88 were detected in human and dog isolates. Our study revealed a high prevalence of faecal ESC-R *E. coli* carriage in both dogs and humans from Romanian households and shelters, where bidirectional clonal transmission between humans and dogs is likely. Furthermore, we identified ESC-R *Enterobacterales* co-carriage in people and dogs sharing the same environment using FTIR, demonstrating its value in AMR surveillance for humans and animals.

## 1. Introduction

Production of extended-spectrum beta-lactamases (ESBLs) remains one of the most important resistance mechanisms to extended-spectrum cephalosporins (ESCs; e.g., cefpodoxime, cefotaxime, ceftazidime, and ceftriaxone) in *Enterobacterales* from both humans and animals [1,2]. In addition, numerous studies over the last two decades have shown that extended-spectrum cephalosporin-resistant (ESC-R) *Enterobacterales* have been isolated with increasing frequency from humans and animals [3,4]. Importantly, the presence of ESBL, AmpC beta-lactamase, and plasmid-mediated quinolone resistance (PMQR) genes on mobile genetic elements facilitates horizontal transmission leading to multidrug resistant (MDR) isolates in both human and animal hosts [2,5]. In addition, ESBL resistance genes also have the potential to spread via successful bacterial clones or lineages (Woodford, 2008; Ewers et al., 2012), such as *Escherichia coli* ST131, which has driven the worldwide dissemination of the CTX-M-15 enzymes [6]. More recently, new high-risk MDR clones, such as *E. coli* D-ST648 and ST410, which combine characteristics of multidrug resistance and virulence, have emerged in companion animals [7,8].

Several studies have described the local prevalence of ESBL-producing *E. coli* in both clinically healthy and diseased companion animals [9,10,11,12], with some reporting high levels of faecal carriage in healthy animals [13,14], highlighting the role that they may play as reservoirs of resistant bacteria. Consequently, there is a concern that the close contact between humans and companion animals could facilitate interspecies transmission of MDR organisms through direct or indirect contact. Although a few studies have reported the prevalence of ESC-R *Enterobacterales* and/or ESBL-producing bacteria in companion animals, the potential for interspecies transmission, defined as the co-carriage of commensal ESC-R or ESBL-producing *E. coli* isolates in people and dogs sharing the same environment, has not yet been fully determined. In addition, studies on antimicrobial resistance (AMR) in companion animals in Romania are sparse, despite it being often placed “in the red zone”, based on the AMR data collected by the European Antimicrobial Resistance Surveillance Network from the European Centre for Disease Prevention and Control (ECDC) [15]. Consequently, there is a gap in the literature that has become particularly important as numbers of domestic cats and dogs have increased significantly in Europe, and notably so in Romania. According to 2021 data published by the European Pet Food Industry, pet dog ownership is very prevalent among Romanian households and one of the highest in Europe, with almost half of the population (42%) owning at least one dog in 2020 [16].

Hence, we aimed to determine the prevalence of ESC-R *Enterobacterales* faecal carriage and co-carriage of identical or different MDR strains in dogs and people sharing a house or shelter environment. Secondly, we aimed to determine the genetic background and clonal relatedness of ESC-R *Enterobacterales* obtained from these hosts and the potential for AMR interspecies transmission using a novel bacterial typing tool.

## 2. Results

### 2.1. Study Groups and Bacterial Isolates

Faecal samples were collected between January 2017 and December 2018 from (i) dogs and owners attending first opinion veterinary practices from North-East, Romania and (ii) dogs and kennel staff from three dog shelters (DSs) from two different geographical regions in Romania [two in North-East (DS-1 and DS-1A) and one in South-East (DS-2). Sampling of the first group was based on dog visits for health checks and, in the second group, was based on random collections of freshly voided faecal samples from individual dog kennels.

Overall, 263 faecal samples from both humans and dogs were collected and analysed. The majority of faecal samples were collected from dogs (*n* = 212), originating from both households (HH, *n* = 102) and dog shelters DS (*n* = 110), whereas 51 samples were collected from humans, from both study groups. For HH samples, dog and owner paired samples were collected from 32 HHs, most of which provided one dog and one owner sample, except for two HHs that submitted three samples, consisting of samples from two dogs and one owner in one case and from two owners and one dog in the other case. Owner samples were not submitted from all the HHs recruited for the study; therefore, only dog samples were obtained from the remaining HHs (*n* = 70). In addition, 129 faecal samples were collected from dog shelters (DSs), where 110 samples were from dogs (DS-1, *n* = 58; DS-1A, *n* = 12; and DS-2, *n* = 40) and 19 faecal samples were collected overall from staff working at the three dog shelters (Figure S1).

Faecal sample screening on Brilliance ESBL selective agar led to the isolation of 104 ESC-R *Enterobacterale*s (104/263, 39.5%) across all sample groups. The prevalence of ESC-R *Enterobacterales* was higher in dogs from households (45/102, 44.11%) compared with those from dog shelters (30/110; 27%). Moreover, 43.7% of owners (14/32) and 78.9% (15/19) of staff working in dog shelters were found to be faecal carriers of these organisms. Among the 70 households (of the 134 HHs) that submitted only dog (therefore, non-paired) faecal samples, 45 (64%) households had dogs that tested positive for ESC-R *Enterobacterales*. Most of the ESC-R isolates were obtained from dogs from DS-2 (14/40, 40%) and DS-1 (15/58, 26%), while no isolates were obtained from DS-1A.

Species determination identified the majority of ESC-R isolates as *E. coli* (94/104, 90.38%), with the remaining isolates identified as *Proteus* spp. (6/104, 5.76%), and *Klebsiella pneumoniae* (4/104, 3.84%). Phenotypic combination disk testing for ESBL-production demonstrated that 19% of dogs (41/212) and 39.21% (20/51) of humans were faecal carriers of ESBL-producing *Enterobacterales*. These mainly comprised *E. coli* (*n* = 57), of which 41 were from dogs and 16 from humans, and *Klebsiella* spp., (*n* = 4), with most isolates (*n* = 3) from dogs.

Antimicrobial susceptibility testing (AST) was performed on all 75 ESC-R *Enterobacterales* from dogs. They showed high resistance to ampicillin (100%), amoxicillin/clavulanic acid (85.71%), aztreonam (73.81%), trimethoprim/sulfamethoxazole (54.76%), enrofloxacin (45.23%), and tetracycline (54.76%). In addition, lower levels of resistance were recorded for gentamicin (23.81%) and chloramphenicol (35.71%). AST was also performed on all ESC-R *Enterobacterales* human isolates (*n* = 29), which also showed variable levels of resistance to ampicillin (95.24%), aztreonam (52.38%), trimethoprim/sulfamethoxazole (57.14%), enrofloxacin (76.19%), tetracycline (71.43%), chloramphenicol (47.62%), gentamicin (47.62%), and amoxicillin/clavulanic acid (19.04%). Nevertheless, all isolates characterized in this study were fully susceptible to imipenem.

### 2.2. Characterization of ESBL and Other Resistance Genes

Seventy-three ESC-R *Enterobacterales* (51 dog and 22 human isolates) were found to carry genes or gene combinations responsible for resistance to extended-spectrum cephalosporins, either by PCR or WGS (Table 1). When screening for beta-lactamase genes in the ESC-R *Enterobacterales* dog isolates, the most prevalent gene was *bla*_CTX-M_ (33/51, 64%), and sequencing identified *bla*_CTX-M-14_ (*n* = 6), *bla*_CTX-M-1_ (*n* = 5), and *bla*_CTX-M-15_ (*n* = 5) as the most common gene variants in dog isolates, followed by *bla*_CTX-M-9_ (*n* = 2) and *bla*_CTX-M-3_ (*n* = 2) (Table 1).

Human isolates also predominantly carried CTX-M group genes (11/22, 50%), with *bla*_CTX-M-14_ (*n* = 3), *bla*_CTX-M-15_ (*n* = 2), *bla*_CTX-M-1_ (*n* = 2), and *bla*_CTX-M-3_ (*n* = 1) found in the sequenced isolates. For both dog and human ESC-R isolates, CTX-M genes were found alone (*n* = 31) or in co-carriage with *bla*_TEM_, *bla*_SHV_, *bla*_OXA_, *qnrB*, or *qnrS* (*n* = 13).

From the AmpC beta-lactamase group genes, only one *bla*_CIT-M_ gene was identified in a dog isolate that co-harboured the *bla*_TEM_ gene; however, more chromosomal AmpC variants (e.g., *ampC1*, *ampH*) were found in isolates undergoing WGS.

When screened for PMQR genes, *qnrS* was the most prevalent gene found in both human (*n* = 4) and dog (*n* = 11) isolates, followed by *qnrB* in one human and three dog isolates, and *aac-(6′)-lb-cr* was found in two human and three dog isolates.

### 2.3. Phylogroup and PCR-Based Replicon Typing

PCR-based phylogroup typing of ESC-R *E. coli* human and dog isolates, (*n* = 94; 66 dog and 28 human isolates) showed that the most prevalent phylogroup for dogs was B1 (24/66; 36.36%), followed by A (19/66; 28.8%), B2 (15/66; 22.72%), and D (8/66; 12.12%). For human isolates, the most prevalent phylogroup was A (11/28; 39.29%), followed by B2 (9/28; 32.14%), D (7/28; 25%), and B1 (1/28; 3,57%). MDR *E. coli* sequence type 131 (ST131) has recently emerged as a worldwide cause of human extraintestinal infections, and more recently, a cause of clinical infections in companion animals. Both host groups have been confirmed as faecal carriers, and so we screened for this clone in our study populations. Three *E. coli* isolates (two human isolates and one dog) from three different households were identified by PCR to belong to the pandemic B2-O25-ST131 clone.

**Figure 1:**
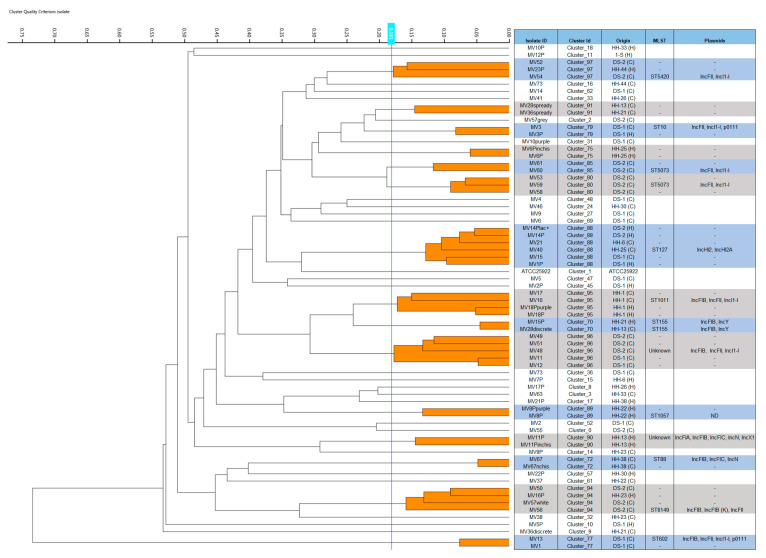
Dendogram obtained by clustering FTIR spectra of *Escherichia coli* extended-spectrum cephalosporin-resistant (ESC-R) faecal isolates (*n* = 69) from healthy dogs, dog owners, or shelter staff. The analysis was based on Euclidean average spectra of three replicates for each isolate; the blue line represents the adjusted cut-off value (COV) of 0.181. Clusters composed of two isolates or more are shaded in orange. The isolate ID, cluster ID, origin, WGS-based MLST, and plasmids, identified by PlasmidFinder, are shown from left to right columns. Rows are shaded in alternate colours (blue and grey), with isolates clustering together highlighted in the same shade. HH = household; DS = dog shelter; H = human; C = canine; ND = not detected.

PCR-based replicon-typing (PBRT) identified multiple replicon types in most *E. coli* ESC-R isolates (*n* = 57), except for several isolates with single replicons (*n* = 18), whereas for six isolates, their plasmid profiles were non-typeable by PBRT. IncFIB was the most prevalent replicon found in both dog and human isolates (*n* = 41), most of which (63%, 26/41) also carried *bla*_CTX-M_ genes. IncFIB was found mostly in combination with IncI1 (*n* = 19), *n* (*n* = 3), Y (*n* = 2), P1 (*n* = 2), L/M (*n* = 2), and A/C (*n* = 1). Most common single replicons belonged to HI2 (*n* = 9), and occasionally IncI2 (*n* = 2), P1 (*n* = 1), and N (*n* = 2). The plasmid replicon types identified by WGS (PlasmidFinder) in fourteen isolates is shown in Figure 1.

### 2.4. Fourier Transform Infrared Spectroscopy (FTIR) Typing, Multi-Locus Sequence Typing (MLST), ESC-R/ESBL Resistance Genes, and Plasmid Cluster Traits

The FTIR spectroscopy analysis is presented in Figure 1, including 69 ESC-R *E. coli* isolates characterised by conventional PCR. Fourteen of these isolates were also investigated by WGS. Overall, fifteen clusters were identified, with two to six isolates per cluster (C_); WGS-based MLST is shown against the tested isolate from each cluster. Six clusters included a mixture of both human and canine isolates, with the representative isolate typed to the following STs: C_70/ST155; C_79/ST10; C_88/ST127; C_94/ST8149; C_95/ST1011; and C_97/ST5420. The remaining clusters included canine isolates only (e.g., C_72/ST88; C_77/ST602; C_85/ST5073; C_91; and C_96, for which the ST could not be determined—ND) or human isolates only (e.g., C_70/ST155; C_72/ST88; C_75/ST ND; and C_89/ST1057). In one case, both isolates from the same cluster (C_70) underwent WGS, which typed them to ST155, showing good agreement between FTIR clustering and MLST typing. Several clusters included (i) epidemiologically related isolates from the same HH (C_95/ST1011; C_89/ST1057; C_72/ST88; C_90/ST ND) or from the same DS (C_79/ST10; C_80 and C_85/ST5073) or (ii) epidemiologically unrelated isolates, either from different HHs (C_70/ST155), from HHs and DSs (C_88/ST127; C_97/ST5420; C_94/ST8149) or different DSs (C_96/ST ND).

Interestingly, some human and dog isolates from HHs were clustered together with shelter isolates by FTIR. For instance, C_88/ST127, which was the largest cluster identified with six isolates, included two ESC-R *E. coli* dog isolates from HH-6 and HH-25 (MV16 and MV17), one dog (MV15) and one staff (MV1P) isolate from DS-1, and two staff isolates from DS-2. All isolates from this cluster (except one) had a common genotype and resistance profile, as they belonged to PG B2 and carried *bla*_CTX-M-1_, *qnrS*, and HI2 plasmids. A similar pattern was seen in C_94/ST8149, where a human isolate from HH-23 clustered together with three dog isolates from DS-2; again, a common genotype was seen in all isolates from this cluster, with all isolates typed to PG A and carrying *bla*_CTX-M-1/15_, *qnrS*, and FIB plasmids. Other occurrences of co-carriage and/or clonal interspecies transmission have been demonstrated by FTIR by clustering together dog and staff isolates from the same shelter (DS-1), such as in C_79/ST10, where both isolates were carrying *bla*_TEM/SHV_ and IncF/I1. In addition, cross-transmission between dogs seemed likely at DS-2, with five dog isolates forming two close FTIR clusters (C_80 and C_85), but all carrying *bla*_CTX-M-1_, IncFII, and IncI1-I. In addition, the representative isolates from these two clusters belonged to ST5073, suggesting that they may have in fact represented the same clone, and the FTIR cut-off value may have been too stringent, separating the isolates into two clusters. Another example of possible dog-to-dog cross-transmission in DS-2 was suggested by C_77/ST602, where both isolates belonged to PG B1 and carried *bla*_CTX-M-14_ and IncFIB/I1. Finally, C_96, (for which the MLST could not be determined) provided an interesting clustering, including five ESC-R *E. coli* dog isolates from both shelters; however, the isolates from the same shelter exhibited identical genotypes, but differed across shelters. Hence, two isolates from DS-1 belonged to PG D and did not carry any of the resistant determinants investigated, whereas three isolates from DS-2 all typed to PG A and carried *bla*_CTX-M-1_, *qnrB*, and FIB/I1.

### 2.5. Co-Carriage of ESC-R Isolates in Dogs and Humans from Households and Dog Shelters

Co-carriage of ESC-R *E. coli* isolates with identical or different molecular characteristics was identified in dogs and humans sharing a household or shelter environment. Of the 32 households that submitted dog-owner paired faecal samples, co-carriage of ESC-R isolates was found in 12 HHs (37.5%); however, only two HHs (6%) found dog and owner ESC-R isolates with identical FTIR spectra, phylogroup, resistance genes, and Inc plasmids. In one of these households (HH1), four ESC-R isolates were obtained (two phenotypically distinct isolates from the owner, and two isolates from his two dogs), which formed a single FTIR cluster (C_95/ST1011), demonstrating likely clonal interspecies transmission within this household. All isolates from this cluster belonged to PG D, carried *bla*_CTX-M-group_-_9_, and Inc FIB/I1 plasmids. The second identical co-carriage was identified in HH-21, where both the dog and owner carried ESC-R *E. coli* of PG B1; however, they lacked the investigated ESBL or FQ resistance genes, likely due to its resistance to ESCs through other (inherent) resistance mechanisms. In the remaining 10 HHs, both humans and dogs were identified as ESC-R carriers; however, they carried isolates with different genotypes and resistance genes. Staff and dog co-carriage of identical phenotypic/genotypic clones was identified twice in DS-1, as demonstrated in C-79/ST10 where one staff member and one dog both carried ESC-R *E. coli* of PG A, *bla*_TEM/SHV_, and IncFII/IncI1. Similarly, in C_88/ST127, one staff member and one dog isolate belonged to PG B2 and carried *qnrS* and Inc HI2; however, the dog isolate also carried *bla*_CTX-M-1_, which was not detected in the human isolate. This last cluster also included two staff isolates from DS-2 with the same genotype (PG A, *bla*_CTX-M-1;TEM/SHV_, and IncFII/IncI1), which also suggests (or does not exclude) direct or indirect human-to-human transmission.

## 3. Discussion

To the best of our knowledge, this is the first study investigating the carriage of ESC-R and ESBL-producing *Enterobacterales* in dogs in Romania, providing first insights into the antimicrobial resistance in companion animals in this country. Data submitted to the European Antimicrobial Resistance Surveillance Network (EARS-Net) in the last few years have shown that, in human isolates, Romania consistently has a high prevalence of invasive bloodstream infections associated with antimicrobial resistance [17]. Despite this concerning record, there is limited information on the prevalence of AMR in bacterial populations from humans [18,19,20,21] and, with the exception of a few studies on the prevalence of *Staphylococcus* spp. in dogs [22,23,24], AMR data in companion animals is lacking. In this study, we found that 44.11% of healthy dogs from households carried ESC-R *Enterobacterales*, which is similar to carriage rates of cefotaxime-resistant *E. coli* isolates found in healthy dogs in the Netherlands (45%) [14], and slightly lower than that reported in dogs from South Korea (49%) [25]. However, the prevalence of ESC-R *Enterobacterales* found in healthy, veterinary-visiting dogs in Romania (44%) is higher than rates recently reported in healthy dogs from France and Spain (18.5 and 19.6%, respectively) [26,27], Greece (22%) [28], and Brazil, Canada, and the US (25, 26.5, and 29%, respectively) [10,13]. At the same time, there are very few studies that has measured the prevalence and molecular characteristics of ESC-R *Enterobacterales* obtained from animal shelters. Umeda et al. (2019) [29] investigated the prevalence of cephalosporin-resistant *Enterobacteriaceae* (CRE) in sheltered dogs and cats from various backgrounds in Japan and found that 14.6% of the dogs and 11% of the cats were carriers of beta-lactamase-producing *Enterobacteriaceae* of high genetic diversity. Another study, which investigated the occurrence of potentially zoonotic and cephalosporin-resistant enteric bacteria in dogs from 10 different shelters in the US, found faecal CRE carriage in 27% of the studied dogs [30]. Furthermore, based on the concern that the increasing number of dogs imported into Finland may represent a source of AMR into the country, which has a low prevalence of AMR in human and dog populations (5 and 6.3%, respectively), Johansson et al. (2022) [31] used WGS to characterize ESBL/AmpC-producing *Enterobacteriaceae* isolated from dogs imported to Finland from several countries, including Romania. Overall, they found the highest prevalence of ESBL-producing *Enterobacteriaceae* in dogs from Russia (55%) and Romania (43%), even though there was a small number of samples from dogs originating from Romania (*n* = 6). In our study, we detected cephalosporin-resistant *Enterobacterales* in 27.27% of the dogs from the shelters, which is likely to be a better reflection of ESC-R carriage in this population given the larger sample size in our study. We also identified 19% of dogs and 39.21% of humans as faecal carriers of ESBL-producing *Enterobacterales*. This is similar to the prevalence reported for ESBL faecal carriage in dogs from Switzerland (17%), but much higher than those reported in dogs from France (3.7%) and the UK (1.9%) [32,33,34]. Although diverse, there was a predominance of *bla*_CTX-M-14_, *bla*_CTX-M-1_, and *bla*_CTX-M-15_ found in ESBL-producing *E. coli* isolated from dogs (64%) and humans (50%) sharing a HH or DS environment in Romania. This is similar to findings from a recent study from Switzerland that identified *bla*_CTX-M-15_ (54.8%) and *bla*_CTX-M-1_ (24.7%) in ESBL-producing *Enterobacteriaceae* from clinical samples of companion animals [32]. The role of plasmids in the dissemination and sharing of *bla*_CTX-M_ between humans and animals has been widely reported [35]. In our study, we found IncFIB to be the most prevalent replicon present in 63% of both dog and human isolates that were carrying *bla*_CTX-M_ genes. This is in contrast to a study that characterized ESBL isolates from pets in France, where IncFII plasmids were mostly associated with *bla*_CTX-M_ genes [34]; however, both plasmid families are recognised for their roles in building antibiotic resistance in *Enterobacteriaceae*.

Although the ESC-R *Enterobacterales* isolates investigated in this study were selected on a medium containing a 3rd generation cephalosporin (cefpodoxime), non-susceptibility to beta-lactam antibiotics was not identified in all isolates when tested against ampicillin or amoxicillin/clavulanic acid. Previous studies have shown that ESBL producers can display variable resistance to penicillin and other beta-lactam antibiotics, depending on the type of enzyme produced and its activity, which can lead to variable susceptibility in vitro (e.g., porin alteration, production of OXA-30 and TEM-1 associated with the loss or alteration of major porins, and outer membrane impermeability) [36].

Surveillance of ESC-R *Enterobacterales* in the community is also of clinical importance, as these organisms are opportunistic pathogens and could be associated with infections that often require higher tier antibiotics, driving resistance to last resort antimicrobials. Only a handful of studies have investigated the prevalence of faecal carriage in people who are dog owners or come in close contact with dogs within a dog shelter environment. A recent longitudinal study performed in the Netherlands examined 550 pairs of human-dog faecal samples and identified a prevalence of ESBL-producing *Enterobacteriaceae* of 3.8% for human participants and 10.6% for dogs [37]. A similar study from South Korea revealed similar findings, with a higher prevalence of ESC-R *E. coli* in dog samples (49.2%) compared with human stool samples (17.3%) [25]. In contrast, a study from New Zealand that focused on the genomic relatedness of ESBL or cephalosporinase-producing *E. coli* in people with community-acquired urinary tract infections (UTIs) and their household members (people and/or pets), identified that the same strain of ESBL-/ESBL-producing *E. coli* was more often cultured from both the UTI index case and another person in the household (5/11 households), than from the household pet (2/11 households) [38].

This is the first study in which FTIR was used to determine the clonal relatedness of ESC-R *E. coli* isolates from dogs and their owners or dog shelter staff, as a means of determining clonal transmission of AMR isolates within a shared environment. FTIR is a phenotypic method that quantifies the absorption of infrared light by a mixture of molecules, particularly the carbohydrates and lipopolysaccharides present in the bacterial cell [39]. When compared with the gold standard techniques for bacterial typing (PFGE and WGS), FTIR spectroscopy shows important advantages, including low costs and short turnaround time (less than 3 h), that make it a promising tool for real-time strain typing. This technology has already been used to investigate hospital outbreaks associated with *K. pneumoniae*, *Enterobacter cloacae*, and other gram-negative bacilli [40]. In addition, several studies have investigated the reliability of clustering between the FTIR and conventional typing methods, such as MLST, pulsed-field gel electrophoresis (PFGE), and whole-genome sequencing (WGS), and have found to high consistency between methodologies. As such, Martak et al. (2019) found that FTIR spectroscopy could accurately cluster together outbreak isolates of *Pseudomonas aeruginosa*, *K. pneumoniae*, and *Enterobacter cloacae* that belonged to the same ST [40,41]. Other studies have compared FTIR clustering results with those obtained by WGS and found high consistency between these methods for clinical or environmental isolates of *E. coli*, *K. pneumoniae*, and *E. cloacae* [42,43,44]. Furthermore, one comparison study found FTIR to have higher discriminatory power than MLST, and no contradictory results between WGS, PFGE, and MLST when compared with FTIR typing [45].

In this study, we used FTIR to investigate the potential interspecies transmission of ESC-R *Enterobacterales* between humans and dogs within a shared environment, and we identified co-carriage of ESC-R isolates with indistinguishable FTIR spectra, resistance profiles, and genetic backgrounds (PG) in both HHs and dog shelters. However, only 6% of paired human-dog faecal samples from HHs yielded co-carriage of an identical strain, which is similar to findings by van den Bunt et al. [37], but lower than findings from Brazil, where 9.5% of the pairs dog/owner included in the study had MDR *E. coli* with identical PFGE profiles [46]. We have identified clonal interspecies or dog-to-dog cross-transmission within both HHs (e.g., C_95/ST1011 and C_70/ST155) and shelter environments (e.g., C_79/ST10, C_88/ST127, C_97/ST5420, C_80, and C_85/ST5073), where transmission events could easily occur through direct or indirect contact. Nevertheless, epidemiologically unrelated human isolates from households and dog isolates from shelters clustered together in the FTIR analysis. The largest cluster of this type of group was C_88/ST127, which included human isolates from the staff of DS-1 and DS-2, as well as isolates from two different HHs. ST127 is recognised as extraintestinal pathogenic *E. coli* (ExPEC), highly adapted for colonising and infecting both humans and companion animals, and a leading cause of urinary tract infections worldwide [47]. The successful transfer of *E. coli* ST127 between humans and dogs have been previously demonstrated [48], and this ST is often considered one of the *E. coli* genotypes with zoonotic potential. Therefore, the high relatedness of ESC-R *E coli* isolates in this cluster that have originated from different hosts, environments, and geographical locations matches the global distribution of ST127. Other important *E. coli* genotypes identified in our study were ST10 (isolated from a staff member and a dog from DS-1) and ST155 (isolated from an owner and a dog from different households), which have been described as major ExPEC lineages with global dissemination [49]. Both were found as commensals in the human and animal intestinal samples, and appeared to have a higher prevalence of plasmid-carried AMR genes, particularly *mcr-1* in ST10, whereas ST155 has been described as a major vector of ESBL genes spread from animals to humans [50]. ST88 (identified in our study in a human from a household), similarly to ST10, is also globally distributed, and previous studies have shown that they are common ESBL-producers circulating between animals and humans [51]. Therefore, given the worldwide distribution of these STs, the identification of ST127, ST10, ST155, and ST88 in human and dog hosts in this study may be considered unsurprising; however, this is the first report of faecal colonisation with *E. coli* of these genetic backgrounds in dog and human populations in Romania.

Our study had some limitations. Although a questionnaire was planned and distributed to the owners and dog shelter staff at enrolment, the rate of survey responses was low and data collection from both HHs and dog shelters could not be completed. Thus, previous antimicrobial exposure or other factors that may have contributed to AMR carriage in the commensal enteric microbiota of the sampled population is unknown, and risk factors for carriage in either dogs or humans included in the study could not be evaluated. Also, we did not perform environmental sampling in this study to determine whether environmental contamination (e.g., dog shelter environment) could have been a cause for the high ESC-R *E. coli* prevalence in dog shelter staff. However, other studies have demonstrated that the environment can act as an AMR reservoir and plays a key role in the dissemination of antimicrobial resistance genes [2].

In conclusion, our study identified a high prevalence of faecal ESC-R *E. coli* in dogs in Romanian households and dog shelters and their human contacts. Moreover, our findings revealed co-carriage of identical and different ESC-R *E. coli* strains in both HHs and dog shelters, where bidirectional clonal transmission between humans and dogs is likely. Furthermore, investigation of clonal transmission is often challenging, and we showed that FTIR was effective in identifying ESC-R *Enterobacterales* co-carriage in people and dogs sharing the same environment, demonstrating its value for AMR surveillance in human and animals.

The free movement of people and their pets within the European Union has been shown to be a risk factor for the translocation of diseases and their vectors [52], and given the recent trend of importing companion animals from Eastern European countries (including Romania), the risk of AMR transmission via these imported animals needs to be evaluated.

## 4. Materials and Methods

### 4.1. Study Population

The aim of the study was to collect faecal samples from human and dogs sharing the same environment and screen them for AMR carriage. The first study group included households (HH) from the North-East of Romania, comprising owners with healthy dogs (kept indoors) visiting the veterinary practice for routine health checks, vaccination, deworming, and flea and tick control. The second group included clinically healthy dogs and kennel staff from three dog shelters (DSs) found in two different geographical regions. Two of these were in the North-East (DS-1 with 200 dogs and DS-1A with 30 dogs) and one (DS-2, housing 60 dogs) in the South-East of Romania. Ethics approval for the project was obtained from the Iasi Faculty of Veterinary Science Ethics Committee. Owners and shelter staff were informed about the aims of the project and informed consent was obtained from all study participants.

### 4.2. Bacterial Isolates

Faecal samples were collected between January 2017 and December 2018 from dogs attending first opinion veterinary practices or the Faculty of Veterinary Science Referral Hospital in Iasi City, Romania. Owners were provided with faecal sampling kits, including Universal Containers with a collection spoon (AlphaLabs, UK), and provided with written instructions to collect the human and dog faecal samples just before a veterinary appointment was due for their pet. Samples were transported to the veterinary practice during the planned visit, which called the laboratory for collection. For the dog shelters, sampling kits including transport swabs were provided in advance for collection of faecal samples (1–2 g) from staff and dogs, and sent to the laboratory.

In the laboratory, faecal samples (1–2 g) were plated out immediately onto Brilliance ESBL Agar (Oxoid, Basingstoke, UK) and incubated at 37 °C for 18–24 h. Brilliance ESBL Agar is a chromogenic selective medium based on a mixture of antibacterial agents, including cefpodoxime, a well-recognised marker for detection of ESBL production, providing presumptive ESBL identification in 24 h. However, other *Enterobacterales* resistant to extended-cephalosporins (ESC-R) can also be selected on this chromogenic medium via non-ESBL resistance mechanisms [53]. ESC-R *Enterobacterales* display characteristic colonies on Brilliance ESBL Agar as follows: blue or pink colonies for *E. coli*; green colonies for *Klebsiella* spp., *Enterobacter* spp., *Serratia* spp., and *Citrobacter* spp.; and tan colonies with a brown halo for *Proteus* spp., *Morganella* spp., and *Providencia* spp. When observed, these colonies were subcultured onto 5% sheep blood agar (SBA; Oxoid, Basingstoke, UK) for further testing. Species identification was performed with biochemical testing (API 20E, Biomerieux) for all *Enterobacterales* and *uidA* or *uspA* genes for *E. coli* isolates was confirmed by PCR [54,55], given the 78% API test accuracy for identification of genera and species [56].

### 4.3. Antimicrobial Susceptibility Testing

All ESC-R isolates that generated characteristic ESBL-like colonies on the Brilliance ESBL Agar (*n* = 104) were subcultured onto SBA and further tested for demonstration of phenotypic ESBL-production with the combination disk test (CDT), including cefpodoxime, ceftazidime, and cefotaxime alone and in combination with clavulanic-acid (MAST Group, United Kingdom). In addition, all *Enterobacterales* selected on the Brilliance ESBL Agar were subject to further antimicrobial susceptibility testing by disk diffusion on Mueller-Hinton agar (MHA), according to the Clinical and Laboratory Standards Institute methodology [57]. The antimicrobial panel included: amoxicillin/clavulanic acid (30 μg), ampicillin (10 μg), aztreonam (30 μg), imipenem (10 μg), trimethoprim/sulfamethoxazole (25 µg), enrofloxacin (5 μg), tetracycline (30 μg), chloramphenicol (30 μg), and gentamicin (10 μg) (all disks and media were from Oxoid, Basingstoke, UK). *E. coli* ATCC 25,922 was used as the control for the disk diffusion susceptibility testing. Interpretation of the antimicrobial susceptibility results was done according to the CLSI [57]. An isolate was considered non-susceptible if it had intermediate or resistant results against the tested antimicrobial agent.

### 4.4. Characterization of Beta-Lactamase and FQ Resistance Genes

All ESC-R isolates (*n* = 104) obtained in this study were analysed for the presence of beta-lactamase and FQ resistance genes by conventional PCR. Freshly grown isolates on SBA were used for DNA extraction by suspending bacterial cells in 500 mL of distilled water and incubating for 10 min at 95 °C, followed by centrifugation for 3 min at 10,000 RPM. The supernatant was used as a template for the PCR assays screening for genes encoding beta-lactamases: *bla*_SHV_, *bla*_TEM_, *bla*_OXA1-like_, and *bla*_CTX-M_, *bla*_ampC_ beta-lactamases, PMQR (*qnrA*, *qnrB*, *qnrS*), and *aac-(6′)-lb-cr* [58,59,60,61]. DNA sequencing was performed on CTX-M amplicons using the same sets of primers as in the original reactions (Eurofins MWG Operon, Germany). PCR amplicons were purified with the Macherey-Nagel NucleoSpin Gel and PCR Clean-up (Thermo Fisher Scientific, Swindon, UK) and nucleotide sequences were compared with those present in GenBank (https://blast.ncbi.nlm.nih.gov/Blast.cgi, accessed on 1 July 2021) to identify the specific gene type or variants involved. *E. coli* isolates carrying resistance determinants associated with ESC and/or FQ resistance were selected for further molecular investigations.

### 4.5. Molecular Characterization of E. coli Genetic Background and PCR-Based Replicon Typing

Characterisation of *E. coli* phylogenetic groups (A, B1, B2, and D) was investigated through multiplex PCR [62]. Furthermore, the isolates typed to phylogenetic group B2 were further tested for the presence of the O25:ST131 clone through simplex PCR, using specific primers for the *rfb.1* gene and for the genes, *trpA* and *pabB* [63]. PCR-based replicon-typing (PBRT) was performed as described by Carattoli et al. [64].

### 4.6. Fourier Transform InfraRed Spectroscopy (FTIR); Sample Preparation and Analysis

ESC-R *E. coli* were selected to assess the clonal relationship between human and animal isolates, which was determined by Fourier transform infrared (FTIR) spectroscopy technology incorporated in the IR-Biotyper (IRBT, Bruker Daltonics GmbH & Co. KG, Bremen, Germany). This is a technique that quantifies the absorption of infrared light by the mixture of molecules present in the sample (e.g., lipids, nucleic acids, carbohydrates, lipopolysaccharides, and proteins), which results in generation of a specific FTIR spectrum, enabling accurate bacterial typing. For FTIR analysis, bacterial isolates (*n* = 69) were grown on 5% sheep blood agar (Oxoid, Basingstoke, UK) for 18 (+/−0.5) hours at 37 °C, followed by extraction, according to the IR Biotyper manufacturer’s instructions. Briefly, bacterial cells were harvested using a 10 μL loopful of bacterial cells and submerged in vials containing 50 μL of 70% ethanol in 1.5 mL vials containing miniature sterile rods for homogenisation. Thorough vortexing was applied for 1–2 min, followed by the addition of 50 μL of molecular grade water to obtain a solution with a final volume of 100 μL. After repeated homogenisation, 15 μL of each suspension was placed in triplicate on a silicon plate (Bruker Daltonics GmbH & Co. KG) and allowed to dry for 30 min at 37 °C. The infrared Test Standards (IRTS1 and IRTS2, Bruker Optics-Daltonics GmbH) were included in each run as controls to confirm that the instrument was calibrated and could discriminate different strains. Three technical replicates were analysed in three independent experiments, using the IR Biotyper system with default analysis settings. Spectra were acquired, visualised, and analysed by OPUS v7.5 software (Bruker Optics GmbH, Ettlingen, Germany). Quality control was performed according to the manufacturer’s recommendations and based on several criteria, such as absorption, noise, presence of water, and fringes.

Dendrograms expressing hierarchical cluster analysis (HCA) were generated by the IR Biotyper Client Software v1.5 (Bruker Daltonics GmbH & Co. KG), using the Euclidian distance and average linkage clustering methods using a cut-off value (COV) of 0.181 to define clusters. This COV was established in our laboratory, prior to analysing the test isolates, using an optimisation procedure where *E. coli* isolates (*n* = 6) that had been previously typed by MLST (some of which were known to be genetically related) were used to determine an optimal COV for *E. coli* specific to our local laboratory conditions. *E. coli* ATCC25922 was also included in the optimisation process. The COV was defined by analysing five replicates of each optimisation isolate in three independent experiments prepared in the same way as for the test isolates; the obtained COV of 0.181 was within the range of COVs recommended by the manufacturers based on user inputs. Hence, a COV of 0.181 was used for cluster analysis, replacing the more generous COV automatically generated by the software algorithm.

### 4.7. Whole Genome Sequencing (WGS) Analysis

To determine the genetic background of the isolates typed by the IR-Biotyper, fourteen ESC-R *E. coli* isolates were selected for WGS analysis. These included isolates representative of different FTIR clusters, and covered isolates from dogs (*n* = 11), including one dog isolate from four different HHs, two dog isolates from DS-1, and five isolates from DS-2, as well as three human isolates (*n* = 3) from different HHs.

Genomic DNA extraction, sequencing, assembly, and annotation of the *E. coli* isolates were provided by MicrobesNG, Birmingham, UK (http://www.microbesng.uk; accessed on 15 August 2021). In brief, DNA libraries were arranged using Nextera XT Library Prep Kit (Illumina, San Diego, CA, USA) and sequencing was performed using an Illumina HiSeq with a 250 bp paired-end protocol. De novo assembly was conducted using SPAdes version 3.7 [65], and contigs were annotated using Prokka 1.11 [66] and re-ordered against the reference *E. coli* K12 genome (GenBank Accession U00096) using MAUVE Alignment Tool [67]. These bacterial sequences were subsequently run through a range of online services in order to obtain the following from the assembled contigs: (i) Multi-locus sequence type (MLST) using *E. coli* 1 scheme [68] plasmid types, offered by the Centre for Genomic Epidemiology at www.cge.cbs.dtu.dk/services/MLST/ (accessed on 1 July 2021) and www.cge.cbs.dtu.dk/services/PlasmidFinder/ (accessed on 1 July 2021), respectively; (ii) Antimicrobial and biocide resistance genes through the Comprehensive Antibiotic Resistance Database (CARD) at http://arpcard.mcmaster.ca (accessed on 1 July 2021) [69].

## Figures and Tables

**Table 1:** Molecular characterisation of extended-spectrum cephalosporin-resistant (ESC-R) *Enterobacterales* in faecal samples from dogs and humans from households and dog shelters. Only isolates that were identified to carry resistance determinants to ESC or FQs are shown in the table. Abbreviations: FTIR, Fourier transform infrared spectroscopy; PBRT, PCR-based replicon typing; PMQR, plasmid-mediated quinolones resistance genes; ND, not detected; NI = not identified; UN, unknown. ^®^ Represents CTX-M-1 group PCR amplicons not sequenced.

Host	Source	Bacterial Species	No. and Isolate ID (*n* = 73)	Cluster by FTIR	PG Typing	MLST by WGS	Beta-Lactamase Genes by PCR	Beta-Lactamase Genes by WGS	PMQR Genes	Additional Resistance Determinants by WGS	Inc Type by PBRT
Human	Dog owner	*E. coli*	1 (MV10P)	18	A	-	CTX-M-1 group^®^	-	*-*	-	FIB; I1
		*E. coli*	1 (MV16P)	94	A	-	CTX-M-1 group^®^	-	*qnrS*	-	FIB
		*E. coli*	1 (MV22P)	57	A	-	*bla*_CTX-M-15_, *bla*_TEM_, *bla*_OXA_	-	*aac-(6′)-lb-cr*	-	FIB/FIA; F
		*E. coli*	1(MV21P)	17	B2	-	*bla*_CTX-M-14_	-	*-*	-	FIB/FIA; F
		*E. coli*	1 (MV18P purple)	95	D	-	*bla*_CTX-M-14_, *bla*_TEM_	-	*-*	-	FIB; F/I1
		*E. coli*	1 (MV11P)	90	B1	UN	*bla*_SHV_, *bla*_TEM_	*bla*_TEM-1_, *bla*_SHV-134_, *ampC1*, *ampH*	*-*	*sul3*, *tet(A)*, *APH(4)-Ia*, *AAC(3)-IV*, *aadA*, *aadA2*, *APH(3′)-Iia*	N
		*E. coli*	1 (MV11P-Inc)	90	D	-	*bla*_SHV_, *bla*_TEM_	-	*qnrB*	-	FIB/FIA/W/K; N/F
		*E. coli*	1 (MV23P)	97	D	-	*bla*_TEM-1_	-	*-*	-	P1
		*E. coli*	1 (MV17P)	8	B2	-	*bla*_OXA_	-	*qnrS;* *aac-(6′)-lb-cr*	-	FIB/FIA; F
		*E. coli*	1 (MV8P purple)	89	B2	ST1057	*ampC*	*ampH*		ND	
		*E. coli*	1 (MV15P)	70	B1	ST155	*ampC*	*ampC1*, *ampH*		*AAC(2′)-IIa*	
	Kennel staff	*E. coli*	1 (MV5P)	10	A	-	*bla*_CTX-M-1_, *bla*_TEM_	-	*-*	-	Y; I1
		*E. coli*	1 (MV14P)	88	B2	-	*bla*_CTX-M-1_	-	*qnrS*	-	HI2
		*E. coli*	1 (MV19P-Inc)	ND	A	-	*bla*_CTX-M-3_	-	*-*	-	FIB; F
		*E. coli*	1 (MV20P-pink)	ND	B2	-	*bla*_CTX-M-15_, *bla*_TEM_	-		-	FIB; F/I1
		*E. coli*	1 (MV24P)	ND	B2	-	CTX-M-1 group, *bla*_TEM_	-	*-*	-	FIB/FIA; L/M
		*E. coli*	1 (MV19P)	ND	D	-	*bla*_CTX-M-14_, *bla*_TEM_	-	*-*	-	FIB; F/I1
		*E. coli*	1 (MV3P)	79	A	-	*bla*_SHV_, *bla*_TEM_	-	*-*	-	F/I1
		*E. coli*	1 (MV12P)	11	D	-	*bla*_TEM-1_	-	*-*	-	FIB; F
		*E. coli*	1 (MV26P)	ND	B2	-	*bla*_TEM-55_, *bla*_OXA-1_	-	*-*	-	FIB/FIA; F
		*E. coli*	1 (MV1P)	88	B2	-	-	-	*qnrS*	-	HI2
		*Klebsiella*	1 (MV4P-Kleb)	ND	ND	-	*bla*_SHV_	-	*-*	-	N
Dog	Households	*E. coli*	1 (MV18)	ND	B2	-	*bla*_CTX-M-1_	-	*qnrS*	-	HI2
		*E. coli*	1 (MV21)	88	D	-	*bla*_CTX-M-1_	-	*qnrS*	-	HI2
		*E. coli*	3 (MV19; MV22; MV40)	ND; ND; 88	B2	ST127 (MV40)	CTX-M-1 group	*bla*_CTX-M-1_	*qnrS*	*qnrS1*, *ampH*, *sul1*, *tet(A)*, *tet(B)*, *APH(3′)-Ia*, *APH(6)-Id*, *APH(3″)-Ib*	HI2
		*E. coli*	1 (MV73)	36	B2	-	CTX-M-1 group^®^, *bla*_TEM_	-	*qnrS*	-	P1; FIB; F
		*E. coli*	1 (MV67)	72	C	ST88	*bla*_CTX-M-3_	*bla*_CTX-M-3_, *ampC1*, *ampH*	*-*	ND	FIB, N
		*E. coli*	1 (MV46)	24	B2	-	*bla*_CTX-M-15_	-	*-*	-	FIB; F
		*E. coli*	1 (MV67-Inch)	72	A	-	*bla*_CTX-M-15_	-	*-*	-	FIB; N
		*E. coli*	1 (MV47)	ND	D	-	*bla*_CTX-M-15_, *bla*_TEM_, *bla*_SHV_	-	*-*	-	F2A; L/M
		*E. coli*	1 (MV39)	ND	B1	-	*bla*_CTX-M-14_	-	*-*	-	FIB; I1
		*E. coli*	3 (MV20; MV66; MV16)	ND; ND; 95	D	ST1011	*bla*_CTX-M-14_; *bla*_TEM_	*bla*_CTX-M-14_, *ampC1*, *TEM-1*	*-*	*sul2*, *AAC(3)-Iid*	FIB; I1
		*E. coli*	1 (MV43)	ND	B1	-	*bla*_CTX-M-9_	-	*-*	-	FIB/K; I1
		*E. coli*	1 (MV17)	95	D	-	*bla*_CTX-M-9_; *bla*_TEM_	-	*-*	-	FIB; I1
		*E. coli*	1 (MV37)	61	B1	-	*bla*_TEM_; *bla*_CIT-M_	-		-	A/C
		*E. coli*	1 (MV29)	ND	B1	-	*bla*_TEM-1_	-	*-*	-	FIB/Y/FIA
		*E. coli*	1 (MV30)	ND	A	-	*bla*_TEM-1_	-	*-*	-	FIB; I1
		*E. coli*	1 (MV33)	ND	A	-	*bla*_TEM-1_	-	*-*	-	FIB; F
		*E. coli*	1 (MV62)	ND	B1	-	*bla*_TEM_	-	*-*	-	FIB
		*E. coli*	1 (MV63)	3	B2	-	*bla*_TEM_	-	*-*	-	-
		*E. coli*	1 (MV38)	32	B1	-	*bla*_SHV_, *bla*_TEM_	-	*-*	-	FIB; I1
		*E. coli*	1 (MV31)	ND	B1	-	*bla*_SHV_, *bla*_TEM_, *bla*_OXA_	-	*-*	-	FIB; N/F
		*E. coli*	1 (MV71)	ND	A	-	*bla*_TEM-1_, *bla*_SHV-52_	-	*-*	-	FIB; F/I1
		*E. coli*	1 (MV69)	ND	B1	-	-	-	*aac-(6′)-lb-cr*	-	FIB; F
		*E. coli*	1 (MV28)	ND	B1	ST155	*ampC*	*ampC1*, *ampH*		AAC(2′)-IIa	
		*Klebsiella*	1 (MV34)	ND	NI	-	*bla*_SHV_, *bla*_TEM_	-	*qnrA*, *aac-(6′)-lb-cr*	-	HI2
		*Klebsiella*	1 (MV71 purple)	ND	NI	-	*bla*_SHV-52_	-	*-*	-	-
		*Klebsiella*	1 (MV68)	ND	NI	-	*bla*_SHV-2_	-	*-*	-	-
	Kennels	*E. coli*	2 (MV53; MV54)	80; 97	A	ST5420	CTX-M-1 group^®^	*bla*_CTX-M-3_, *ampH*	*-*	ND	F/I1
		*E. coli*	3 (MV59; MV60; MV61)	80; 85; ND	B1	ST5073	CTX-M-1 group^®^	*bla*_CTX-M-3_, *ampC1*	*-*	ND	F/I1
		*E. coli*	1 (MV15)	88	B2	-	*bla*_CTX-M-1_	-	*qnrS*	-	HI2
		*E. coli*	1 (MV50)	94	A	-	*bla*_CTX-M-1_	-	*qnrS*	-	FIB
		*E. coli*	1 (MV55)	0	B1	-	*bla*_CTX-M-1_, *bla*_TEM_	-	*qnrS*	-	I1
		*E. coli*	1 (MV56)	94	A		CTX-M-1 group^®^		*qnrS*		FIB
		*E. coli*	3 (MV48; MV49; MV51)	96; 96; 96	A		CTX-M-1 group^®^		*qnrB*		FIB; I1
		*E. coli*	1 (MV58)	80	B1		*bla*_CTX-M-3_		*-*		F/I1
		*E. coli*	1 (MV52)	97	A		*bla*_CTX-M-15_		*-*		F; I1
		*E. coli*	1 (MV57)	2	A		*bla*_CTX-M-15_		*qnrS*		FIB
		*E. coli*	2 (MV1; MV13)	77; 77	B1		*bla*_CTX-M-14_		*-*		FIB; I1
		*E. coli*	1 (MV3)	79	A		*bla*_SHV_, *bla*_TEM_		*-*		F/I1
		*E. coli*	1 (MV4)	48	A		*bla*_SHV_, *bla*_TEM_		*-*		P1; FIB; Y; N/I1
		*E. coli*	1 (MV9)	27	A		*bla*_SHV_, *bla*_TEM_		*-*		I1
		*E. coli*	1 (MV14)	62	B2		*bla*_SHV_, *bla*_TEM_		*aac-(6′)-lb-cr*		FIB

## Data Availability

The main datasets generated and analysed as part of this study are included within the manuscript.

## Supplementary Materials

The following supporting information can be downloaded at: https://www.mdpi.com/article/10.3390/antibiotics11091242/s1, Figure S1: Faecal sample origin and extended-spectrum cephalosporin-resistant (ESC-R) isolates obtained from dogs and humans sharing the same household or animal shelter environment in Romania.
