# Supplementary figures and images for: Clonal Dissemination of Extended-Spectrum Cephalosporin-Resistant Enterobacterales between Dogs and Humans in Households and Animal Shelters of Romania
Source: Antibiotics (Basel). 2022 Sep 13;11(9):1242. doi: 10.3390/antibiotics11091242 (PMC9495119; doi:10.3390/antibiotics11091242)

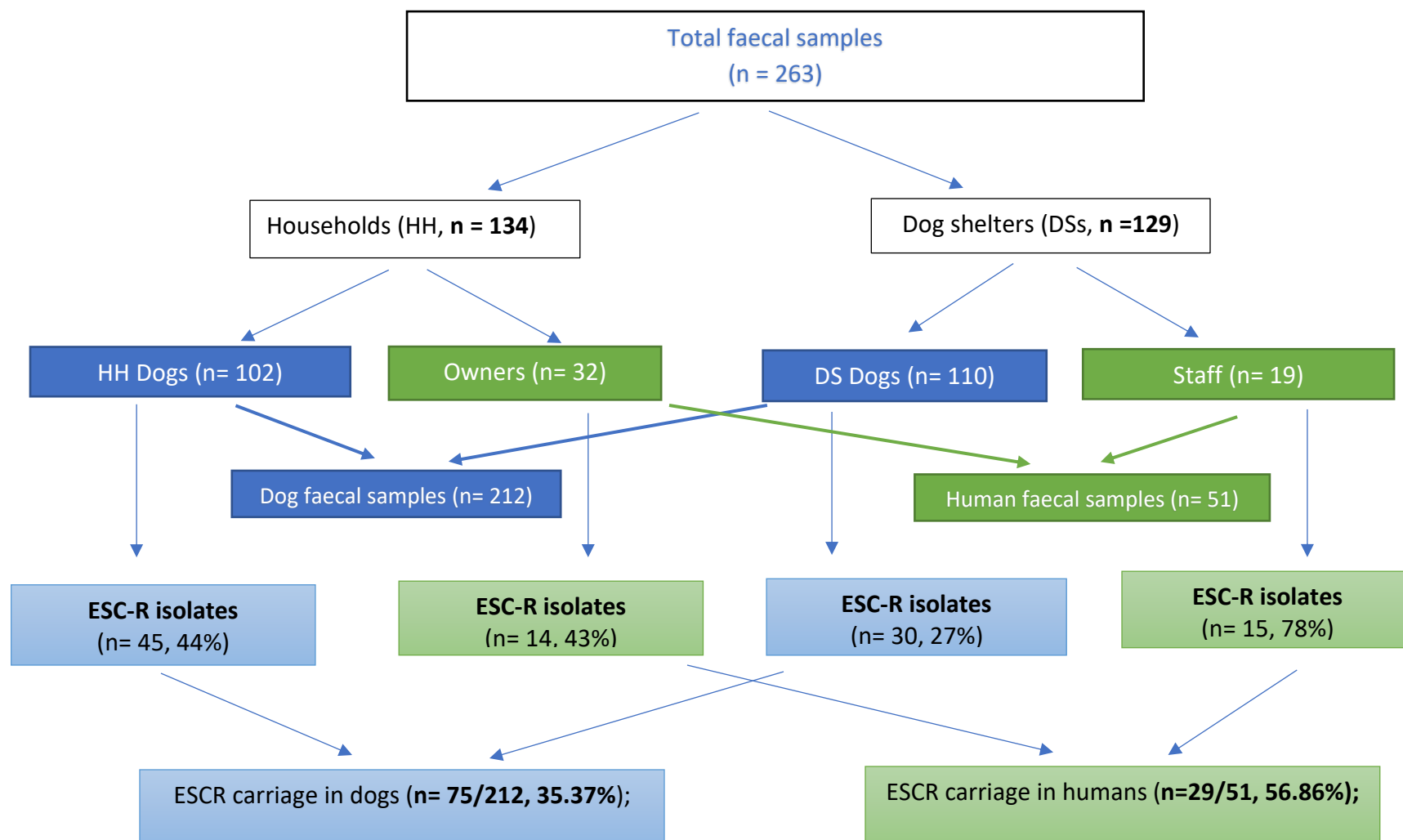

Supplement: Supplementary file 1 [file antibiotics-11-01242-s001.zip › antibiotics-1884834-supplementary.pdf]
